# Occupational Health and Performance Among Chinese University Teachers: A COR Theory Model of Health-Promoting Leadership and Burnout

**DOI:** 10.3390/ejihpe15070134

**Published:** 2025-07-14

**Authors:** Xiaohua Sha, Yulin Chang

**Affiliations:** 1 Chinese International College, Dhurakij Pundit University, Bangkok 10210, Thailand; shaxiaohua@ncmc.edu.cn; 2 Department of Research, Nanchang Medical College, Nanchang 330052, China; 3 Department of Educational Psychology and Counseling, National Taiwan Normal University, Taipei 106308, Taiwan

**Keywords:** health-promoting leadership, job burnout, job performance, conservation of resources theory, occupational health, university teachers

## Abstract

With the rapid expansion of higher education in China, university teachers are facing increasing workloads and mounting performance pressures, posing significant threats to their occupational health. Consequently, how to enhance job performance while safeguarding faculty well-being has become a critical issue for higher education administrators. This study aims to explore the role of health-promoting leadership (HPL) in addressing the dual challenge of enhancing university teachers’ job performance while maintaining their occupational health. Drawing on the Conservation of Resources (COR) theory, this study conceptualizes job burnout as both a core indicator of occupational health and a mediating variable, as well as proposing a dual-path model to examine the direct and indirect effects of HPL on teachers’ job performance. A survey of 556 university teachers in Jiangxi Province, China, was conducted; the data were analyzed using IBM SPSS Statistics version 22.0 and AMOS version 26.0 (IBM Corp., Armonk, NY, USA). The findings suggest that HPL is positively associated with job performance, both directly and indirectly through reduced burnout, supporting a dual-pathway mechanism consistent with COR theory. These results contribute to a better understanding of the potential role of HPL in balancing teacher well-being and performance in the context of Chinese higher education. This study also extends the cross-cultural application of COR theory and provides theoretical and practical insights into how HPL may help alleviate teacher burnout and support the development of health-promoting universities.

## 1. Introduction

With the fast-paced advancement of higher education in China, the responsibilities of university teachers have become increasingly multifaceted, encompassing teaching, research, service, and administrative duties. These roles are not only complex and intensive but also highly performance-driven, contributing to mounting levels of occupational stress and psychological strain among faculty members (Sun et al., 2023). Haddon (2018) emphasized that sustained work pressure constitutes a considerable risk to teachers’ health and well-being, particularly in competitive and resource-constrained environments. This has been associated with a rising incidence of job burnout, anxiety, and depression in the academic profession (Razak, 2019).

Recent studies point out the intensification of “involution” in Chinese universities, with teachers averaging 51.64 working hours per week and over 50% being categorized as heavy overworkers (J. Yang et al., 2024). Such prolonged overwork seriously compromises both physical and mental health. A meta-analysis by R. Yang et al. (2019) further revealed a steady decline in the psychological well-being of Chinese university faculty members in recent years, negatively impacting their professional growth and overall quality of life. Against this backdrop, occupational health has become a critical issue in higher education.

Importantly, occupational health is not only related to individual well-being but is also closely tied to job performance. The performance of university faculty members is crucial for ensuring the quality of teaching and the success of students (Huang et al., 2022). As the core of the academic system, university teachers directly impact institutional outcomes through their involvement in education, research, and administration (Jimenez, 2021), and they play a key role in the sustainable development of higher education institutions (Jadeja, 2022).

Research by Dooris et al. (2020) further suggests that safeguarding the health and well-being of university teachers is a prerequisite for enhancing their performance. Psychological stress and occupational burnout can impair teachers’ ability to fulfill their core responsibilities. Burnout is characterized by emotional exhaustion, depersonalization, and a decline in personal accomplishment, reflecting an individual’s maladaptive response to long-term work stress (Alwaely & Jarrah, 2020). Persistent burnout severely damages both psychological health and professional performance, leading to decreased engagement, increased absenteeism, and even premature departure from the workforce (Mourad et al., 2025). The negative impacts of these factors directly affect employees’ job performance and career satisfaction (Alwaely & Jarrah, 2020; Mourad et al., 2025).

This study is grounded in the Conservation of Resources (COR) theory, which posits that when individuals face resource depletion, their job performance and psychological health are negatively affected. According to COR theory, the emergence of burnout stems from the depletion of an individual’s resources and the inability to effectively recover, leading to emotional exhaustion and depersonalization among teachers in the workplace. Therefore, reducing burnout is crucial for long-term occupational health and also contributes to improving teachers’ job performance (Hur et al., 2024).

In recent years, an increasing number of studies have shown that health-promoting leadership (HPL) plays an important role in enhancing employee well-being and performance (Furunes et al., 2018; Nielsen & Taris, 2019). According to the COR theory, HPL helps alleviate personal resource depletion by providing supportive resources, restoring psychological health, and improving work performance (Yao et al., 2021). In high-pressure environments, HPL offers emotional support, improves the work climate, allocates resources effectively, and fosters a supportive and caring atmosphere. These actions reduce work-related stress, significantly lower the risk of burnout, protect occupational health, and enhance employee performance (Peng et al., 2022).

In the field of education, the World Health Organization and UNESCO (2021) introduced global standards aimed at transforming every school into a health-promoting institution. HPL is seen as a key driving force behind this transformation. In recent years, the Chinese government has increasingly emphasized public health. The “Healthy China 2030” plan advocates for a comprehensive health strategy, requiring the integration of this concept into various fields such as education, healthcare, and urban governance (Bai et al., 2022). However, empirical research on HPL in China, particularly in higher education, remains limited (W. Liu et al., 2020; Q. Wang et al., 2021). Notably, the mechanisms through which HPL impacts teacher performance, as well as the potential mediating role of occupational health, have not been fully explored.

Therefore, this study, grounded in COR theory, explores whether an HPL style, centered on employee well-being, supportive communication, and providing work-related resources, can enhance university teachers’ job performance. It also examines whether burnout, as a core indicator of occupational health, moderates the relationship between HPL and job performance. **This study proposes a psychosocial resource-based framework to understand leadership effectiveness in higher education and its role in balancing teachers’ occupational health and job performance.**

To guide the investigation, the following research questions are proposed:

Q1: How does HPL influence job burnout among university teachers?

Q2: What is the impact of job burnout on university teachers’ job performance?

Q3: What is the relationship between HPL and job performance among Chinese university teachers?

Q4: Does job burnout mediate the relationship between HPL and job performance?

## 2. Literature Review

### 2.1. Health-Promoting Leadership (HPL) and Job Burnout

Job burnout is a persistent occupational syndrome characterized by emotional exhaustion, depersonalization, and a diminished sense of personal accomplishment (Maslach & Leiter, 2016). It reflects a state in which individuals feel physically and mentally depleted, demotivated, and unable to remain engaged in their professional roles (Bakker & de Vries, 2021).

Burnout is widely recognized as a serious occupational health issue, particularly in the education sector. Research has shown a significant negative correlation between teacher burnout and mental health, with emotional exhaustion and psychological stress commonly impacting teachers’ quality of life and teaching effectiveness (Giri & Paria, 2023; Kumari & Prajapati, 2024). Moreover, job stress has been found to intensify burnout, significantly increasing turnover intentions, especially in higher education settings (Pei et al., 2024).

Recent research has conceptualized burnout as a dynamic psychological process, not merely a static outcome. Schaufeli et al. (2020) emphasized that burnout arises not only from high workloads but also from an enduring imbalance between invested effort and gained resources. In emotionally demanding professions such as teaching, chronic resource loss without sufficient replenishment can initiate a “loss spiral” that exacerbates psychological fatigue over time.

The COR theory, introduced by Hobfoll (1989), provides a compelling framework for understanding this phenomenon. According to COR theory, individuals strive to acquire, retain, and safeguard resources—such as emotional energy, time, and social support—to cope with stress. When resource loss outweighs resource gain, especially over prolonged periods, the risk of burnout intensifies (Hobfoll, 2001; Prapanjaroensin et al., 2017). While COR theory has been widely applied in occupational health research, its adoption within higher education—particularly in China—remains limited (H. Wang, 2024).

In China, university faculty are increasingly vulnerable to burnout due to continuous pressure from teaching, research, administrative tasks, and service obligations (Y. Yang & Ling, 2023). High workloads combined with insufficient institutional support systems significantly elevate psychological distress and reduce job satisfaction (Gao et al., 2024; P. Wang et al., 2020). Therefore, strategies aimed at resource preservation and replenishment are critical for safeguarding faculty well-being.

Kariou et al. (2021) and Y. Wang and Kee (2025) further underscore the importance of organizational context, showing that burnout is closely associated with emotional demands and a lack of perceived support. Cross-cultural research also suggests that burnout patterns vary by cultural values, leadership norms, and power distance, highlighting the necessity for localized strategies in non-Western academic environments (Korkmazyurek & Ocak, 2024).

HPL is one such strategy. HPL refers to a leadership style that actively promotes employee well-being through health-oriented behaviors such as expressing care, reducing workplace stressors, and fostering a supportive work environment (Kaluza et al., 2020). Its central goal is to enhance employee health and resilience, thereby improving organizational outcomes (Franke et al., 2014).

Recent empirical studies have demonstrated that HPL is significantly negatively associated with job burnout (Arnold & Rigotti, 2021; Horstmann, 2018). For example, Klebe et al. (2021) and Vonderlin et al. (2020) found that caring leadership reduces employee fatigue and psychological strain, contributing to lower levels of burnout in knowledge-intensive professions. In the field of higher education, HPL has been shown to reduce teacher burnout by fostering a supportive climate wherein educators feel valued and cared for (Arnold & Rigotti, 2021).

In summary, the existing research confirms the value of HPL in reducing job burnout, especially in knowledge-intensive occupations. However, there is still a lack of systematic evidence regarding how HPL operates within the Chinese higher education context. In particular, studies integrating COR theory with HPL and job burnout remain scarce. This study aims to address this gap by clarifying how HPL, through a resource-conservation mechanism, impacts university teachers’ occupational health by alleviating burnout.

Based on the above, the following hypothesis is proposed:

**H1.**  *HPL has a significant negative effect on university teachers’ job burnout*.

### 2.2. Job Burnout and Job Performance

Job burnout refers to a state of physical, emotional, and psychological exhaustion resulting from prolonged overwork in high-intensity job environments (Schaufeli & Greenglass, 2001). Internationally, burnout has long been a significant focus of research due to its widespread impact on employee well-being and organizational performance.

While the Maslach Burnout Inventory (MBI) has historically been the most commonly used tool for measuring burnout, increasing attention in recent years has been given to the Bergen Burnout Inventory (BBI). Developed by Salmela-Aro et al. (2011), the BBI has been widely applied in European academic research. It conceptualizes burnout as comprising three interrelated dimensions—exhaustion, cynicism, and inadequacy, each reflecting a distinct psychological response to work-related stress.

In the education sector, teachers are especially vulnerable to burnout due to prolonged exposure to heavy teaching loads, administrative pressures, and performance evaluation demands, all of which lead to various negative outcomes (Belay et al., 2023). In China, burnout has become particularly prevalent among university faculty, with complex and far-reaching causes and consequences (Y. Yang & Ling, 2023). Given that Chinese university teachers also face high workloads and limited access to supportive resources, the BBI’s emphasis on dimensions such as inadequacy is particularly relevant. This makes the BBI both theoretically compatible and practically suitable for use in the Chinese higher education context.

Job performance is a multidimensional construct that refers to the effectiveness with which employees carry out their work responsibilities. It includes both task-related behaviors and broader organizational contributions. Specifically, job performance captures the quality and quantity of work required to fulfill essential duties (Koopmans et al., 2013), as well as contextual activities such as helping colleagues, participating in institutional initiatives, and demonstrating organizational citizenship (Yoo & Kim, 2021). In contrast, it also involves counterproductive work behaviors—voluntary actions that negatively affect the organization—such as tardiness, absenteeism, or resistance to authority (Kim et al., 2020).

Job performance has long been a central concern in organizational psychology, given its direct relevance to both individual and institutional success. While traditionally evaluated through managerial ratings or output-based indicators, the growing need for comprehensive, behaviorally grounded instruments has led to the development of more nuanced tools. One such widely validated measure is the Individual Work Performance Questionnaire (IWPQ) developed by Koopmans et al. (2013). The IWPQ offers a multidimensional framework for assessing employee performance, incorporating not only task execution but also interpersonal and counterproductive aspects of workplace behavior.

Specifically, the IWPQ conceptualizes job performance as comprising three distinct dimensions, as follows: Task performance, referring to how effectively teachers execute core job responsibilities such as course delivery, curriculum development, and research output. Contextual performance, which includes discretionary behaviors that support organizational climate and functioning—such as mentoring students, assisting colleagues, and contributing to departmental goals (Yoo & Kim, 2021). Counterproductive work behavior, encompassing actions that directly or indirectly hinder organizational functioning, including withdrawal, resistance to policy, and negligent behavior (Kim et al., 2020).

The Individual Work Performance Questionnaire (IWPQ) has demonstrated strong psychometric properties across a range of occupational settings, including education, healthcare, and public service (Jasiński et al., 2023; Lousã et al., 2024). Its emphasis on behavioral indicators rather than subjective impressions makes it particularly suitable for evaluating job performance in knowledge-intensive professions like university teaching. Furthermore, its balanced inclusion of positive (task and contextual) and negative (counterproductive) behaviors enables a more holistic view of faculty performance, capturing not only teachers’ strengths but also their areas of potential concern.

The three dimensions of occupational burnout—emotional exhaustion, cynicism, and diminished professional efficacy—have been shown to negatively impact all facets of job performance. Chronic emotional exhaustion impairs both energy levels and cognitive functioning, thereby reducing task efficiency and hindering academic productivity. Cynicism, marked by detachment and a negative attitude toward work, diminishes interpersonal engagement and cooperation, ultimately weakening contributions to the organizational context. Diminished professional efficacy, reflecting a loss of confidence and motivation, further undermines an individual’s ability to maintain consistent and effective professional output (Gamarra et al., 2023; Palenzuela et al., 2019).

Additionally, job burnout contributes to a decline in occupational health, which refers to the overall mental and physical well-being of employees in the workplace. Poor occupational health—driven by prolonged stress, inadequate recovery, and lack of supportive leadership—has been linked to lower productivity, higher absenteeism, and decreased organizational commitment (Gao et al., 2024). In Chinese higher education, many teachers report psychological distress, sleep issues, and somatic symptoms linked directly to job strain, further affecting their ability to meet performance expectations (L. Yang & Yang, 2025).

Studies show that occupational health serves as both a direct predictor and an indirect mediator of job performance. In particular, positive health status is associated with higher levels of focus, resilience, and adaptive functioning, which are essential for complex, knowledge-based tasks such as university teaching and research (Zhu & Ye, 2022). Therefore, burnout, as a core indicator of deteriorating occupational health, disrupts not only work-related behaviors but also the fundamental psychological conditions that support high performance. As burnout intensifies, teachers reduce effort, lose confidence, and disengage cognitively from their responsibilities—leading to a self-reinforcing cycle of lower performance and increased strain (Corbeanu et al., 2023; Lemonaki et al., 2021).

Given the increasing pressure in China’s higher education sector and the severe depletion of faculty members’ personal resources, understanding how job burnout—as a core indicator of occupational health—affects job performance is essential for promoting the sustainable development of universities. Based on the reviewed literature, we propose the following hypothesis:

**H2.**  *Job burnout has a significant negative effect on university teachers’ job performance*.

### 2.3. HPL and Job Performance

HPL is a leadership style in which leaders actively foster employee well-being and performance by showing care, reducing work-related stressors, and supporting both physical and mental health needs (Kaluza et al., 2020). The core aim of HPL is to create a resource-enriching work environment that not only protects employee health but also promotes sustained organizational functioning (Franke et al., 2014).

Franke et al. (2014) argued that health-promoting leaders influence performance not only by directly providing support but also by serving as role models for healthy behavior, thereby encouraging employees to maintain better self-care routines and psychological balance. This leadership style has been linked to fewer stress-related complaints and improved mental health outcomes across various organizational settings (Klebe et al., 2021; Vonderlin et al., 2020). When implemented effectively, HPL can enhance job performance by mitigating stress and reinforcing motivational and relational resources.

Lichtenthaler and Fischbach (2018) conducted an empirical study showing that employee-oriented leadership significantly improves task performance, proactive performance, and adaptive performance by promoting promotion-focused job crafting. While the study did not directly examine HPL, the concept of employee-oriented leadership shares strong theoretical overlap with HPL, thus providing partial empirical support for the mechanisms by which HPL may influence performance.

Yao et al. (2021), through a systematic review, constructed a conceptual model of HPL, and proposed that HPL can indirectly enhance job performance by enriching employee resources, stimulating positive psychological states, improving health, and strengthening motivation. Although this review lacks empirical testing, it offers a solid theoretical foundation for future research on the HPL–performance relationship.

In the education sector, HPL has also been shown to positively influence teacher performance. Kwatubana (2022) found that HPL fosters a healthy school culture and supportive interpersonal relationships, which significantly improve teachers’ psychological well-being and work engagement. Similarly, Hadijah (2024) reported that leadership styles emphasizing health, values, and support can enhance teacher motivation and instructional commitment, thereby improving task performance and organizational citizenship behaviors.

In summary, HPL functions not only as a health management mechanism but also as a promising driver of employee performance. Existing research shows that HPL can enhance innovative performance, task performance, and work engagement through mechanisms such as resource enhancement, improved well-being, and the activation of proactive behaviors. This effect has been preliminarily confirmed in educational contexts. However, there remains a lack of direct empirical studies examining how HPL influences job performance among university faculty specifically. Based on the above theoretical and empirical foundation, this study proposes the following hypothesis:

**H3.**  *HPL has a significant positive effect on university teachers’ job performance*.

### 2.4. The Mediating Role of Job Burnout

The COR theory posits that individuals are intrinsically motivated to acquire, protect, and sustain valued resources—such as energy, psychological resilience, and health—in order to preserve functional performance and overall well-being (Hobfoll, 1989, 2001). In high-demand professional environments such as universities, sustained resource loss that outweighs resource gain renders employees increasingly vulnerable to stress and, eventually, to job burnout. Burnout, in turn, impairs both the ability and motivation to uphold performance standards, particularly when resource depletion is not counterbalanced by sufficient support or replenishment.

Within the higher education context, burnout arises when faculty members are subjected to chronic work overload, inadequate recovery periods, and limited access to coping resources. According to COR theory, such prolonged strain leads individuals to psychologically disengage from their professional roles as a means of conserving their remaining resources. This disengagement manifests as diminished work effort and declining job performance (Hobfoll, 2001). Conversely, when supportive workplace conditions are established—such as flexible working arrangements, empathetic leadership, and manageable job demands—faculty experience lower levels of burnout and demonstrate greater work engagement (Firdaus et al., 2023; Prentice & Thaichon, 2019).

HPL functions as a resource-enhancing mechanism that mitigates workplace stressors through psychological support, the promotion of healthy behaviors, and the cultivation of a positive work climate (Javaid et al., 2023; Kwatubana, 2022). Leader-provided resources—such as emotional support, clear role expectations, and autonomy—help shield university faculty from the detrimental effects of occupational overload. By alleviating burnout, HPL indirectly supports improvements in job performance across task-related responsibilities, contextual contributions, and reductions in counterproductive behaviors (Diana et al., 2023; Freire et al., 2020).

Recent empirical findings reinforce the mediating role of burnout in the relationship between HPL and job performance. For example, Diana et al. (2023) demonstrated that burnout significantly mediated the link between leadership support and teaching performance. Similarly, Karnia (2023) found that reductions in emotional exhaustion not only enhanced individual concentration but also fostered interpersonal collaboration within university environments. These findings align with prior research indicating that burnout disrupts cognitive functioning, emotional regulation, and social sensitivity—all of which are essential to effective performance in higher education institutions.

In the context of Chinese higher education, the need to understand this mediation pathway is particularly urgent. Recent studies confirm that burnout symptoms among Chinese university teachers are rising sharply, with emotional exhaustion and resource depletion becoming common complaints (Pei et al., 2024). Within such a resource-strained system, HPL may serve as a protective buffer that reduces burnout and indirectly enhances job performance.

Taken together, theoretical and empirical findings suggest that burnout is a key explanatory mechanism through which HPL improves faculty performance. This leads to the following hypothesis:

**H4.**  *Job burnout mediates the relationship between HPL and university teachers’ job performance*.

**Figure 1:**
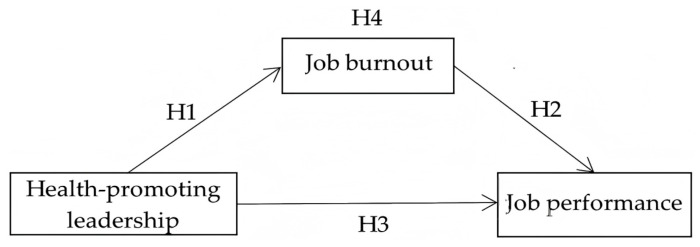
Hypothesized model diagram.

Finally, to visually illustrate the mechanism proposed in this study under the framework of the COR theory, we constructed a hypothesized structural model that clearly depicts both the direct and indirect pathways between HPL and job performance. As shown in Figure 1, HPL is expected to enhance university teachers’ job performance not only directly but also indirectly by reducing job burnout. As a core indicator of psychological resource depletion, job burnout plays a mediating role that reflects the mechanism of resource replenishment and restoration—namely, that supportive leadership behaviors help alleviate teachers’ resource exhaustion, thereby improving their work effectiveness.

## 3. Materials and Methods

### 3.1. Research Design

This study employed a quantitative cross-sectional survey design to investigate the relationship between health-promoting leadership (HPL), job burnout, and job performance among university teachers in China. Data were collected through self-administered questionnaires distributed via an online survey platform.

### 3.2. Research Subjects

This study focuses on university teachers in China. The term refers not only to full-time faculty engaged primarily in teaching and research (Ma et al., 2022) but also to those who hold dual academic and administrative roles within institutional governance. The latter group plays a critical role in coordinating educational resources and advancing institutional development (Han et al., 2020). Jiangxi Province, located in central China, was selected as the survey region because its higher education system is at a mid-level stage of development, making it a representative case that reflects the average conditions of China’s broader higher education landscape.

We employed convenience sampling for questionnaire distribution. Ghiselli et al. (1981) suggested that the sample size should exceed 10 times the number of questionnaire items, and Sudman’s (1976) guidelines suggest 500–1000 participants for regional research; according to this, we determined our target sample size. With 35 questionnaire items in our study, we needed a minimum of 500 valid responses. Accounting for potential non-responses, we distributed 600 questionnaires. Using Meade and Craig’s (2012) criteria, we excluded invalid responses (incomplete answers, identical responses to all items, and questionnaires completed too quickly). Ultimately, 556 valid questionnaires were gathered, corresponding to an effective response rate of 92.67%.

Among the 556 valid respondents, 272 were male (48.9%) and 284 were female (51.1%). The age distribution was balanced: 146 were aged 35 or younger (26.3%), 147 were aged 36–45 (26.4%), 150 were aged 46–55 (27.0%), and 113 were aged 56 or older (20.3%). Participants represented multiple disciplines, including social sciences, engineering, medicine, and education.

Given the use of convenience sampling, potential selection bias should be acknowledged. Caution is warranted when generalizing the findings. Future studies may employ stratified or random sampling to enhance representativeness and external validity.

This study was conducted in accordance with the ethical principles outlined in the Declaration of Helsinki. Participation was entirely voluntary, anonymous, and non-invasive. A detailed informed consent statement was provided to all respondents before data collection, clearly explaining the study’s purpose, data usage, risks, and rights, including the right to withdraw at any time. No sensitive personal or medical information was collected, and this study involved no vulnerable populations or experimental interventions, qualifying as minimal risk social research. Ethical approval for this study was granted by the Ethics Committee for Behavioral Sciences, Social Sciences, and Humanities, Dhurakij Pundit University, Thailand (protocol code DPU_BSH 1110/2567; date of approval 11 October 2024).

### 3.3. Research Instruments

#### 3.3.1. HPL

HPL was measured using the 6-item Health-Oriented Leadership Scale developed by Lutz et al. (2023). Items assessed aspects such as healthcare, occupational safety, work–life balance support, recognition, and health hazard reduction. Sample items include the following: “My leader cares about my health,” “cares about my occupational safety,” “supports me in achieving optimal work–life balance,” “gives me deserved recognition,” and “takes all possible measures to reduce health hazards at school.” Responses were given on a 7-point Likert scale. The scale demonstrated excellent internal consistency (Cronbach’s α = 0.963), with AVE = 0.811 and CR = 0.963. Confirmatory factor analysis showed good model fit (see Appendix A).

#### 3.3.2. Job Burnout

Job burnout was assessed using the 9-item Bergen Burnout Inventory (Salmela-Aro et al., 2011), which evaluates exhaustion, cynicism, and inadequacy. Sample items include the following: “I am snowed under with work” and “I feel dispirited at work and I think of leaving my job.” Participants responded on a 6-point Likert scale. The scale showed good reliability (Cronbach’s α = 0.872), with AVE values of 0.642 and CR values all exceeding 0.840. CFA indicated good model fit (see Appendix A).

#### 3.3.3. Job Performance

Job performance was measured using the 18-item Individual Work Performance Questionnaire (Koopmans et al., 2013), which includes three dimensions: task performance, contextual performance, and counterproductive work behavior. Items were rated on a 5-point frequency scale, with reverse scoring applied to CWB items. Sample items include the following: “I managed to plan my work so that it was done on time” and “I complained about unimportant matters at work.” The scale showed excellent internal consistency (Cronbach’s α = 0.907), with AVE values above 0.500 and CR values above 0.840. CFA confirmed a good model fit (see Appendix A).

### 3.4. Data Analysis

Statistical analyses were conducted using SPSS and AMOS. CFA was used to assess the measurement model, including the validity of the HPL, job burnout, and job performance constructs. Discriminant validity was evaluated by comparing the square root of the AVE values to the inter-construct correlations. Descriptive statistics were calculated to summarize demographic variables and the distributions of key constructs.

To detect potential common method bias (CMB), Harman’s single-factor test was performed. The first factor accounted for less than 40% of the total variance, suggesting that CMB was not a major concern. Pearson correlation analysis was conducted to explore the relationships among the main variables.

To test the structural relationships among HPL, job burnout, and job performance, structural equation modeling (SEM) was conducted. In addition, the mediating role of job burnout was tested using a non-parametric bootstrapping approach with 5000 resamples, as recommended by Nevitt and Hancock (2001). A mediation effect was considered statistically significant if the 95% confidence interval (CI) did not contain zero (Shrout & Bolger, 2002). This method provided robust evidence for the indirect effect of HPL on job performance through reduced burnout.

## 4. Results

### 4.1. Descriptive Statistical Analysis

A descriptive statistical analysis of the main variables revealed that HPL in Jiangxi universities scored above the midpoint (M = 4.243, SD = 1.625), indicating a moderately high level. Job burnout among university teachers was below the midpoint (M = 3.073, SD = 1.033), suggesting a moderately low level of burnout. Job performance scores were also above the midpoint (M = 3.293, SD = 0.717), indicating moderately high performance. All variables demonstrated normal distribution, with skewness and kurtosis values within acceptable ranges (absolute values < 2), as shown in Table 1.

### 4.2. Common Method Bias Test

As data were collected using convenience sampling from a single source, we tested for common method bias using Harman’s single-factor test on all 33 items from the three scales. The KMO value was 0.919 (>0.800), and Bartlett’s test of sphericity was significant (*p* < 0.001), confirming the appropriateness of factor analysis.

Unrotated principal component analysis extracted seven factors with eigenvalues greater than 1, with the first factor explaining only 27.507%, which is well below the 40% indicative threshold (Podsakoff & Organ, 1986). As shown in Table 2, these results indicate that common method bias is unlikely to be a significant issue in this study.

To further address potential common method bias, we compared the fit indices of a seven-factor model with those of a single-factor model. The seven-factor model fit significantly better than the single-factor model, with a significant chi-square difference (Δχ2 = 6205.035, Δdf = 21, *p* < 0.001). As shown in Table 3, these results further suggest that common method bias is not a major concern (Podsakoff et al., 2003).

### 4.3. Correlation Analysis

Pearson’s correlation analysis was conducted to examine the relationships among the main variables. As shown in Table 4, a negative correlation was found between HPL and job burnout (*r* = −0.285, *p* < 0.001), showing initial support for hypothesis H1. Job burnout was negatively correlated with job performance (*r* = −0.289, *p* < 0.001), and so H2 received preliminary support. A positive correlation was observed between HPL and job performance (*r* = 0.313, *p* < 0.001), giving preliminary support for hypothesis H3. All correlation coefficients were below 0.700, indicating no serious multicollinearity issues.

Discriminant validity was evaluated by comparing the square root of AVE with inter-construct correlations. As shown in Table 5, each construct’s square root of AVE (diagonal values) exceeded its correlations with other constructs, confirming sufficient discriminant validity (Fornell & Larcker, 1981).

### 4.4. Structural Equation Modeling Analysis

Following Hair et al.’s (2012) recommendations, we conducted path analysis to test the hypothesized relationships in our research model. The overall model demonstrated excellent fit with the data, as follows: *χ*^2^/*df* = 1.126, GFI = 0.983, AGFI = 0.974, NFI = 0.987, RFI = 0.984, CFI = 0.999, IFI = 0.999 (all exceeding the recommended threshold of 0.900), SRMR = 0.020, and RMSEA = 0.015 (both below the 0.080 threshold).

**Figure 2:**
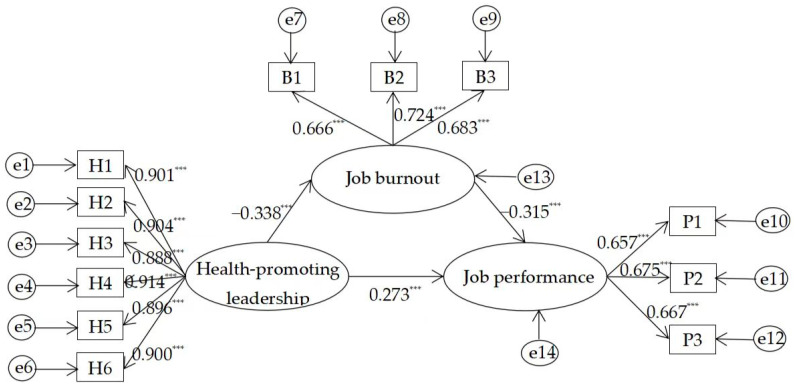
Standardized path diagram of the structural equation model. Note: H1-H6 represent the six items of the HPL scale. B1: Exhaustion. B2: Cynicism. B3: Inadequacy at work. P1: TP. P2: CP. P3: CWB (reverse-scored). *** *p* < 0.001.

The path analysis results, presented in Table 6 and Figure 2, support all three hypotheses. HPL had a significant negative effect on job burnout (*β* = −0.338, *p* < 0.001, 95% CI [−0.431, −0.238]), supporting Hypothesis 1. Job burnout had a significant negative effect on job performance (*β* = −0.315, *p* < 0.001, 95% CI [−0.438, −0.190]), supporting Hypothesis 2. HPL had a significant positive effect on university teachers’ job performance (*β* = 0.273, *p* < 0.001, 95% CI [0.162, 0.379]), supporting Hypothesis 3.

Additionally, we reported the R^2^ values for the endogenous variables. The model accounted for 11.4% of the variance in job burnout (*R*^2^ = 0.114) and 23.1% of the variance in job performance (*R*^2^ = 0.231), indicating the moderate explanatory power of the proposed mediation model (Cohen, 1988).

Figure 2 presents the standardized path diagram of the hypothesized structural equation model. For a complete representation of the modeled relationships, the following equations specify the unstandardized structural relations based on AMOS output:

Job Burnout = −0.182 × HPL + ζ1

Job Performance = 0.104 × HPL − 0.223 × Job Burnout + ζ2

These equations reflect the predictive paths among the latent variables. The error terms (ζ1 and ζ2) represent unexplained variance in the corresponding endogenous constructs.

Following Nevitt and Hancock’s (2001) recommendations, we conducted a bootstrap analysis with 5000 resamples to examine the direct, indirect, and total effects in our mediation model, providing a robust test of the mediating role of job burnout. As shown in Table 7, HPL demonstrated a significant direct effect on job performance (*β* = 0.104, *p* < 0.001, 95% CI [0.061, 0.152]), further confirming our first hypothesis (H1). The analysis also revealed a significant indirect effect through job burnout (*β* = 0.041, *p* < 0.001, 95% CI [0.023, 0.066]), supporting our fourth hypothesis (H4) that job burnout mediates the relationship between HPL and job performance. The total effect was also significant (*β* = 0.145, *p* < 0.001, 95% CI [0.106, 0.187]).

To enhance the interpretability of the mediation, we computed the Proportion Mediated (PM), which is calculated as the ratio of the indirect effect to the total effect (PM = 0.041/0.145 = 0.28). This indicates that 28% of the total effect of HPL on job performance is transmitted via job burnout, representing a meaningful partial mediation pathway (Preacher & Kelley, 2011). Since both the direct and the indirect effects were significant, these results indicate a partial mediation effect.

Taken together, the significant direct and indirect effects indicate that job burnout partially mediates the relationship between HPL and job performance. These findings empirically support the theoretical model proposed in this study, demonstrating that job burnout significantly mediates the relationship between HPL and university teachers’ job performance. HPL not only directly enhances job performance but also operates through an indirect pathway by reducing job burnout levels. This dual-pathway mechanism highlights the importance of HPL in educational settings, offering valuable insights for educational administrators seeking to improve both teacher well-being and performance outcomes.

## 5. Discussion

### 5.1. Health-Promoting Leadership (HPL) and Its Association with Teacher Burnout

The findings of this study reveal that HPL has a significant negative effect on job burnout among Chinese university teachers, with a path coefficient of −0.338 (*p* < 0.001), indicating that leadership behaviors oriented toward employee well-being may be associated with reduced burnout through stress mitigation. For example, Javaid et al. (2023) demonstrated that health-specific leadership and peer social support significantly reduced burnout in emotionally demanding work environments, primarily by lowering perceived stress. Rather than simply presenting statistical outcomes, these results point to a broader relational pattern—when leaders foster psychological resources such as emotional support, stress management, and a caring climate, employees may experience lower levels of emotional exhaustion and disengagement.

The theoretical foundation for these findings can be explained by the COR theory (Hobfoll et al., 2018) and the Stress-Buffering Model of social support. According to COR theory, when individuals perceive the availability of adequate resources in the workplace, they are more able to manage stress and avoid further resource depletion. In this context, HPL functions as a replenishing resource by offering psychological, informational, and task-related support—particularly in resource-intensive environments such as higher education. Through ongoing attention to teachers’ mental and physical well-being, and the cultivation of a supportive and open climate, leadership may help alleviate the psychological impact of high workloads, research pressures, and administrative duties.

The present findings align with the conceptualization of burnout as a process of resource depletion, rather than merely a response to workload demands (Demerouti et al., 2001). By illustrating how leadership behaviors may contribute to resource restoration, this study offers theoretical and applied insights into preventive approaches to burnout. Specifically, leadership support may trigger a “resource gain–recovery–reinvestment” process that contributes to improved emotional resilience and work engagement, which are linked to reduced burnout over time (Tian & Guo, 2022).

In addition, the Stress-Buffering Model suggests that social support serves as a psychological buffer in high-pressure environments. For instance, Santa Maria et al. (2020) found that in high-stress professions such as healthcare, HPL reduced the adverse impact of workload on mental health. Similarly, when teachers perceive leadership behaviors such as fair workload allocation, wellness-oriented feedback, and empathetic communication, they may report greater emotional stability, self-efficacy, and psychological adaptability. These factors, in turn, are associated with lower emotional exhaustion and professional disengagement—two core components of burnout (Fernet et al., 2012).

Moreover, this study adds to the existing literature by empirically examining these associations in the Chinese higher education context—an environment often marked by hierarchical leadership and high emotional labor. This contributes to cross-cultural occupational health research by suggesting that resource-oriented leadership models such as HPL may be relevant in high power-distance, non-Western cultural settings. As such, the findings help address an identified gap in cross-cultural investigations of leadership and occupational well-being.

### 5.2. The Association Between Job Burnout and Teacher Performance in Higher Education

The results of this study indicate that job burnout significantly and negatively affects job performance among Chinese university teachers, with a path coefficient of −0.315 (*p* < 0.001), thus confirming Hypothesis H2. This finding aligns with previous studies suggesting that burnout is related to lower task efficiency and reduced workplace engagement (Karnia, 2023). Burnout—typically defined by emotional exhaustion, depersonalization, and reduced personal accomplishment—may be linked to decreased energy, weakened concentration, and lower levels of responsibility. Over time, these symptoms are often accompanied by professional disengagement, psychological detachment, and potentially counterproductive work behavior.

As a central indicator of occupational health, job burnout reflects a prolonged psychological response to sustained work stress and insufficient recovery. Prior research has associated burnout with increased risks of anxiety, depression, and chronic fatigue (Klusmann et al., 2023). These conditions may impair essential cognitive and emotional capacities, which are critical for teaching quality, academic productivity, and collaboration. For example, Klusmann et al. (2023) observed that burnout is associated with reduced attentional capacity, responsiveness to student needs, and ability to perform cognitively demanding tasks—factors that may affect both individual functioning and broader institutional outcomes.

In the Chinese higher education context, where faculty are typically responsible for teaching, research, service, and administrative tasks, the demands are particularly high. When institutional recovery mechanisms are insufficient, faculty may be more likely to experience a “high demand–low recovery” cycle, which could contribute to psychological resource depletion and diminished performance.

These findings are consistent with the Conservation of Resources (COR) theory (Hobfoll et al., 2018), which posits that individuals seek to preserve and replenish personal resources. When perceived resource loss exceeds perceived resource gain, stress levels rise and functional capacity may decline. In this context, burnout may be viewed as an indicator of sustained net resource loss that is associated with lower professional performance.

Teaching also requires considerable emotional labor, including empathy, emotional regulation, and interpersonal engagement. Once psychological resources are depleted, it may become more difficult for teachers to maintain instructional quality, job satisfaction, and commitment to their institutions. Kariou et al. (2021), for instance, found a negative association between emotional exhaustion and teaching satisfaction and effectiveness among Greek educators. The present study extends this observation to the Chinese higher education sector, where empirical research on the relationship between burnout and performance remains relatively limited.

Taken together, these results suggest that job burnout may be an important factor associated with decreased teacher performance. By examining this relationship within a high-demand, non-Western educational environment, this study contributes to a broader understanding of occupational health challenges in academia. Additionally, the findings expand the limited empirical literature concerning how burnout may influence professional functioning in Chinese higher education institutions.

### 5.3. HPL and Its Association with Teacher Performance

This study found that HPL has a significant positive effect on job performance among Chinese university teachers, with a path coefficient of 0.273 (*p* < 0.001), thereby confirming Hypothesis H3. While Lichtenthaler and Fischbach (2018) examined employee-oriented leadership more broadly, rather than just HPL specifically, their findings pointed to similar mechanisms—suggesting that leadership styles focused on employee well-being may help reduce work-related stress and emotional exhaustion, which in turn is associated with improved engagement and performance.

The present findings are consistent with this view; in higher education settings, when leaders actively support faculty well-being, teachers appear better equipped to manage occupational stress and health-related risks. This may enable them to allocate more psychological energy toward core professional responsibilities such as teaching, research, and service, therefore contributing to enhanced performance outcomes (Y. Liu et al., 2023; Luh et al., 2025).

These results are also supported by COR theory (Hobfoll et al., 2018), which posits that individuals are motivated to conserve and acquire resources to cope with stress and maintain performance. Psychological resources—such as emotional support, perceived autonomy, and control—serve as both protective and enabling factors. When faculty perceive leadership that facilitates access to these resources, they may become more resilient to work demands and more capable of effective professional functioning.

HPL may activate such a resource gain process through various mechanisms; by promoting healthy behaviors, encouraging open and empathic communication, and supporting autonomy, it may help reduce emotional strain and strengthen internal motivation. These supportive dynamics are particularly important for sustaining long-term professional engagement in cognitively and emotionally demanding roles such as academia (Huang et al., 2022).

In China’s competitive academic environment—where quantitative performance indicators are increasingly emphasized—HPL may serve as a counterbalancing force. By addressing emotional and psychological needs, HPL has the potential to buffer against resource depletion and foster greater resilience. This approach may support the alignment of high performance standards with faculty well-being, thereby contributing to more sustainable institutional development.

Overall, the findings support a positive link between HPL and teacher performance, while also highlighting the potential strategic value of adopting health-oriented leadership practices. Integrating such leadership into university management frameworks may help create supportive environments where both well-being and performance are mutually reinforced.

### 5.4. Job Burnout as a Mediator Between Health-Promoting Leadership and Teacher Performance

The present study reveals that job burnout plays a statistically significant mediating role in the relationship between HPL and job performance among Chinese university teachers. Specifically, the direct effect of HPL on job performance was 0.104 (*p* < 0.001), the indirect effect via job burnout was 0.041 (*p* < 0.001), and the total effect was 0.145 (*p* < 0.001). Approximately 28% of the total effect was mediated by burnout, suggesting a partial mediation model and supporting Hypothesis H4. These findings indicate that HPL may influence teacher performance not only through direct pathways but also indirectly by reducing levels of burnout, providing empirical support for the role of burnout as a psychological mechanism linking leadership behavior to performance outcomes.

Burnout, as a central indicator of occupational health, reflects the accumulated strain experienced by faculty in high-demand environments. In situations where emotional or institutional support is lacking, teachers may report higher levels of burnout, which in turn can lead to emotional fatigue, cognitive overload, and reduced engagement. HPL, by fostering emotional support, autonomy, and a psychologically safe climate, may help to reduce this strain and support faculty functioning (Santa Maria et al., 2020).

From the perspective of COR theory (Hobfoll et al., 2018), the mediating role of burnout illustrates how resource-oriented leadership may help interrupt a cycle of resource loss, emotional stress, and performance decline. When psychological and emotional resources are made available through leadership, teachers are more likely to manage stress effectively and maintain productivity.

In addition, reducing burnout may contribute to sustained work engagement and internal motivation, which are important for maintaining performance over time (Y. Yang & Ling, 2023). These insights suggest that burnout should not be viewed solely as a health-related concern, but also as a factor relevant to professional effectiveness and institutional outcomes.

Taken together, the findings highlight that HPL may promote teacher performance through two complementary pathways—directly, as well as indirectly by alleviating burnout. This reinforces the potential value of incorporating well-being-focused leadership strategies into higher education management. Promoting HPL behaviors may therefore serve both humanistic and performance-enhancing functions.

## 6. Conclusions and Implications

### 6.1. Theoretical Implications

First, this study provides empirical evidence supporting the relevance of health-promoting leadership (HPL) within the Chinese higher education sector, thereby contributing to the cross-cultural understanding of HPL theory. While most existing research has focused on Western corporate environments, the present findings suggest that HPL may also be applicable in non-Western, high-power-distance contexts characterized by emotional labor and hierarchical organizational structures (Javaid et al., 2023). This extends the contextual validity of health-oriented leadership models to more diverse institutional settings.

Second, this study contributes to theoretical perspectives on job burnout by conceptualizing it as a dynamic psychological mechanism rather than a static outcome. As a partial mediator in the relationship between leadership and performance, burnout is shown to function both as an indicator of occupational health and as a potential pathway through which leadership influences work outcomes. This dual role aligns with the Conservation of Resources (COR) theory, which emphasizes the protective function of leadership in preventing resource loss through emotional, cognitive, and structural support. Within this framework, burnout emerges as a key construct connecting leadership practices with broader organizational functioning.

Third, by validating a dual-path model in which HPL influences job performance both directly and indirectly through reduced burnout, this study clarifies one potential mechanism by which health-oriented leadership may contribute to sustainable professional outcomes. These findings refine existing theoretical frameworks that integrate leadership, resource preservation, and performance, particularly in roles involving high emotional and cognitive demands, such as academic work.

Finally, this research contributes to the field of occupational health psychology by addressing a noted theoretical gap, namely, the limited availability of explanatory models that describe how leadership can support both employee health and job performance in high-demand educational contexts. By integrating COR theory with research on HPL and burnout, this study offers a theoretically grounded platform for future investigations into psychosocial risk management and professional development in academic institutions.

### 6.2. Practical Implications

This study offers several practical insights for enhancing occupational health and job performance in the higher education sector, particularly within high-demand, resource-constrained environments such as Chinese universities.

First, the findings highlight the potential value of positioning HPL as a strategic management approach rather than an auxiliary well-being initiative. In high-pressure academic contexts, HPL may serve dual functions—supporting faculty well-being and contributing to improved performance outcomes. University leadership may consider incorporating HPL-related competencies—such as empathic leadership, stress-sensitive communication, and health-conscious decision-making—into professional development and leadership training programs. This perspective aligns with emerging research showing that leadership approaches emphasizing psychological safety and emotional support are associated with lower burnout and greater employee engagement (Javaid et al., 2023). Such efforts may be particularly important in light of rising mental health concerns, burnout prevalence, and faculty attrition in Chinese higher education.

Second, this study underscores the importance of recognizing burnout as both a health concern and a potential performance barrier, suggesting that proactive identification and targeted intervention may be beneficial. Institutions are encouraged to monitor indicators of burnout—such as emotional exhaustion and disengagement—and provide structured support systems, including counseling, peer support, and workload balancing. Santa Maria et al. (2020) emphasized the role of proactive strategies in mitigating burnout within high-stress occupations, and the current findings extend this rationale to the academic sector. Developing well-being monitoring systems may help prevent the escalation of burnout and foster long-term faculty engagement.

Third, the mediating role of burnout observed in this study reinforces the relevance of adopting a resource-based approach to academic personnel management. By investing in the emotional and psychological resources of teaching staff, institutions may help break the cycle of resource depletion and performance decline. Recommended practices include flexible work arrangements, promoting autonomy in teaching and research, and encouraging transparent communication between leadership and faculty. These strategies are in line with COR theory findings suggesting that resource reinforcement can serve both protective and performance-enhancing functions (Hobfoll et al., 2018; Lichtenthaler & Fischbach, 2018). Such approaches not only support mental well-being but may also strengthen professional purpose and institutional commitment.

Finally, the findings are consistent with broader policy trends advocating integrated health promotion in educational settings. In alignment with China’s “Healthy China 2030” strategy and the WHO–UNESCO Health-Promoting Schools framework, there may be merit in embedding HPL principles at the institutional level within universities. This is supported by Bai et al. (2022), who argue for the incorporation of health strategies into education governance as a means of strengthening long-term institutional resilience. Operationalizing this vision could include integrating health considerations into performance evaluations, departmental culture, and strategic planning processes. Such efforts may help universities simultaneously support faculty well-being and enhance institutional sustainability and competitiveness.

### 6.3. Limitations and Future Research Directions

Despite its contributions, this study has several limitations that should be acknowledged and may inform future research efforts.

First, the sample was limited to public universities in Jiangxi Province, China, which may constrain the external validity of the findings. Differences in regional governance, institutional capacities, and educational practices could shape how HPL is perceived and implemented. In addition, leadership expectations and cultural norms may vary across provinces and institutional types. Future studies may benefit from recruiting more geographically diverse samples or using stratified sampling methods to enhance generalizability. Cross-cultural comparative research may also help clarify the role of cultural factors—such as power distance or collectivism—in moderating the impact of HPL.

Second, the use of a cross-sectional design limits the ability to draw causal inferences. Although the hypothesized relationships are grounded in COR theory and supported by prior empirical evidence, the temporal sequence among variables remains uncertain. Future research could employ longitudinal or experimental designs—such as leadership intervention studies—to explore how changes in HPL behaviors influence burnout and performance over time. Such approaches may yield stronger evidence of causality, especially in dynamic educational contexts characterized by policy reforms and workload intensification.

Third, the present study focused on job burnout as a mediator, positioning it as a central indicator of occupational health. However, other psychological mechanisms—such as psychological capital, emotional regulation, or professional identity—may also play mediating or moderating roles in the relationship between leadership and performance. In addition, contextual factors at the organizational level—such as perceived institutional support, leadership fairness, or workload equity—may influence the effectiveness of HPL. Future research may consider using moderated mediation models or multilevel frameworks to explore how HPL functions across individual and organizational contexts. Subgroup analyses (e.g., junior vs. senior faculty, or different academic disciplines) may also reveal important variations in effect pathways or boundary conditions.

### 6.4. Conclusions

This study investigated the relationships between HPL, job performance, and job burnout among Chinese university teachers, positioning burnout as a key indicator of occupational health. The results indicate that HPL is significantly and positively associated with teacher performance. This association appears to follow two pathways—a direct positive relationship and an indirect relationship mediated by lower levels of burnout. These findings are consistent with a dual-pathway mechanism grounded in COR theory.

In the context of Chinese higher education—where academic staff are increasingly burdened by teaching evaluations, research quotas, and administrative responsibilities—occupational burnout has become a widespread issue linked to challenges in both faculty well-being and institutional effectiveness. This study contributes to the theoretical understanding of leadership as a potential resource-enriching function. The findings suggest that health-oriented leadership practices are associated with lower burnout and better professional performance under high-stress conditions, thereby enriching the cross-cultural application of HPL and COR theory within the educational sector.

From a practical perspective, the findings highlight the potential value of university administrators developing and promoting HPL behaviors, which include emotional care, fair workload distribution, and open communication. Building a psychologically supportive and resource-abundant work environment may therefore be a key strategy for alleviating faculty burnout and enhancing performance. Such efforts could help advance the strategic goal of creating health-promoting universities that align institutional excellence with educator well-being.

Nevertheless, it is important to acknowledge that due to the cross-sectional nature of this study, the relationships identified reflect associations rather than causal effects. Future research employing longitudinal designs or experimental methodologies would be valuable in establishing the causal pathways suggested by these findings. Additionally, intervention studies testing the implementation of health-promoting leadership practices would provide stronger evidence for the practical recommendations emerging from this research.

## Figures and Tables

**Table 1:** Descriptive statistics of key variables.

Variable	Mean	SD	Skewness	Kurtosis
HPL	4.243	1.625	0.015	−1.222
Job Burnout	3.073	1.033	0.187	−0.911
Job Performance	3.293	0.717	0.104	−1.134

**Table 2:** Results of Harman’s single-factor test.

Factor	Eigenvalue	Variance Explained (%)	Cumulative Variance (%)
1	9.077	27.507	27.507
2	4.128	12.510	40.017
3	3.423	10.374	50.391
4	2.165	6.560	56.951
5	1.762	5.339	62.290
6	1.278	3.871	66.162
7	1.150	3.486	69.648

**Table 3:** Comparison of model fit indices.

Model	*χ* ^2^	*df*	*χ*^2^/*df*	Δ*χ*^2^	Δ*df*	*p*
Single-factor model	6841.999	495	13.822	6205.035	21	<0.001
Seven-factor model	636.964	474	1.344			

**Table 4:** Correlation matrix of main variables (*n* = 556).

Variable	Mean	SD	1	2	3
1. HPL	4.243	1.625	1		
2. Job Burnout	3.073	1.033	−0.285 ***	1	
3. Job Performance	3.293	0.717	0.313 ***	−0.289 ***	1

Note: *** *p* < 0.001.

**Table 5:** Discriminant validity assessment (*n* = 556).

Variable	1	2	3	4	5	6	7
1	**0.901**						
2	−0.227 ***	**0.** **801**					
3	−0.230 ***	0.478 ***	**0.** **801**				
4	−0.233 ***	0.452 ***	0.501 ***	**0.7** **97**			
5	0.267 ***	−0.177 ***	−0.178 ***	−0.160 ***	**0.** **727**		
6	0.261 ***	−0.216 ***	−0.174 ***	−0.146 ***	0.443 ***	**0.** **725**	
7	0.216 ***	−0.217 ***	−0.235 ***	−0.189 ***	0.437 ***	0.453 ***	**0.7** **32**

Note: Bold diagonal numbers show the square root of the AVE. 1: HPL. 2: Exhaustion. 3: Cynicism. 4: Inadequacy at work. 5: TP. 6: CP. 7: CWB (reverse-scored); *** *p* < 0.001.

**Table 6:** Path analysis results.

Path	Unstandardized Coefficient (*B*)	Standardized Coefficient (*β*)	*p*-Value	95% Confidence Interval	
				Lower	Upper
HPL → Job burnout	−0.182	−0.338	<0.001	−0.431	−0.238
Job burnout → Job performance	−0.223	−0.315	<0.001	−0.438	−0.190
HPL → Job performance	0.104	0.273	<0.001	0.162	0.397

Note: *R*^2^ = 0.114 for job burnout and *R*^2^ = 0.231 for job performance, indicating the proportion of variance explained by the model for each endogenous variable.

**Table 7:** Bootstrap mediation effect analysis.

Path	Effect Size	*p*-Value	95% Confidence Interval	
			Lower	Upper
Direct effect	0.104	<0.001	0.061	0.152
Indirect effect	0.041	<0.001	0.023	0.066
Total effect	0.145	<0.001	0.106	0.187
Proportion Mediated (PM)	0.280	-	-	-

Note: Direct effect: HPL → Job performance. Indirect effect: HPL → Job burnout→ Job performance. Total effect: HPL → Job performance.

## Data Availability

The data are available upon request.

## Abbreviations

The following abbreviations are used in this manuscript:

HPL	health-promoting leadership
COR	Conservation of Resources
CFA	Confirmatory Factor Analysis
AVE	Average Variance Extracted
CR	Composite Reliability
TP	Task Performance
CP	Contextual Performance
CWB	Counterproductive Work Behavior

## Appendix A. Measurement Model Fit Indices

Confirmatory Factor Analysis (CFA) Model Fit Indices

Health-Promoting Leadership: *χ*^2^/*df* = 3.819, RMR = 0.017, GFI = 0.995, AGFI = 0.987, NFI = 0.997, IFI = 0.999, TLI = 0.999, CFI = 0.999, RMSEA = 0.009.

Job Burnout: *χ*^2^/*df* = 1.481, RMR = 0.038, GFI = 0.986, AGFI = 0.974, NFI = 0.985, IFI = 0.995, TLI = 0.993, CFI = 0.995, RMSEA = 0.029.

Job Performance: *χ^2^*/*df* = 1.799, RMR = 0.039, GFI = 0.996, AGFI = 0.943, NFI = 0.949, TLI = 0.973, CFI = 0.977, RMSEA = 0.038.
